# Polymorphism in STAT4 Increase the Risk of Systemic Lupus Erythematosus: An Updated Meta-Analysis

**DOI:** 10.1155/2022/5565057

**Published:** 2022-04-22

**Authors:** Shancui-zheng, Jinping-Zhang, Guoyuan-lu, Lei Liu, Zhiyong-deng

**Affiliations:** ^1^Department of Nephrology, The First Affiliated Hospital of Soochow University, Suzhou 215006, China; ^2^Institutions of Biology and Medical Sciences, Soochow University, Suzhou 215006, China; ^3^Department of Rheumatology and Clinical Immunology, The First People's Hospital of Kunshan, Suzhou 215300, China

## Abstract

Previous studies have reported that STAT4 rs7574865 conferred the susceptibility to systemic lupus erythematosus (SLE). In this study, a meta-analysis (including 32 comparative studies of 11384 patients and 17609 controls) was conducted to investigate the role of STAT4 polymorphism in SLE in a comprehensive way. We found that the Asian population had the highest prevalence of the T allele than any other study population at 32.2% and that STAT4 rs7574865 polymorphism was associated with SLE in the overall population (OR = 1.579, 95%CI = 1.497-1.665, *P* < 0.001). In the subgroup analysis by ethnicity, STAT4 rs7574865 T allele was shown to be risk factor in SLE in Asian, European, and American origins. Our results do support STAT4 rs7574865 polymorphism as a susceptibility factor for SLE in populations of different ethnic and that its prevalence is ethnicity dependent.

## 1. Introduction

Systemic lupus erythematosus (SLE) is classified as an inflammatory autoimmune disease characterized by production of autoantibodies to nuclear antigens and immune complex formation [1], leading to multiple organs and systems damage, such as lupus nephritis, lupus encephalopathy, interstitial lung disease, and damage of blood system. Although the etiology of SLE still remains not completely clear, it was generally known that genetic and environmental factors interacting with each other contribute to SLE risk [2].

Genome-wide association studies have identified signal transducer and activator of transcription4 (STAT4) as a novel susceptibility gene associated with SLE [3]. STAT4 consists of 24 exons that spread over a 120 kb region on chromosome 2q32.3, and T is the mutation allele. It encodes a transcription factor that transmits signals. The main STAT4 activating cytokines are IL-12 and IL-23, leading to T-helper type1 and T-helper type17 differentiation with IFN-𝛾 and IL-17 production, which are critical players in the pathogenesis of SLE [4, 5].

As the investigation varied greatly among studies, the association between STAT4 rs7574865 polymorphism and SLE risk has reported mixed results [6–26], which may due to small sample sizes, leading to inadequate statistical power, or interstudy inconsistencies, such as ethnic differences. Therefore, to overcome the limitations of these individual studies and resolve the observed inconsistencies, an exhaustive meta-analysis was performed to reach a credible conclusion.

## 2. Materials and Methods

### 2.1. Identification of Eligible Studies

We conducted a comprehensive literature search for available articles in which STAT4 polymorphism were analyzed in SLE patients, using the databases of PubMed, Web of Science, CNKI (Chinese National Knowledge Infrastructure), and Wanfang. The relevant articles were retrieved by keyword combinations as follows: “STAT4,” “Signal transducer and activator of transcription 4,” “rs7574865,” “polymorphism,” and “systemic lupus erythematosus.” The references in the obtained studies were also examined to identify additional studies not included by the electronic databases, with no language or country restrictions.

### 2.2. Criteria for Inclusion

The inclusion criteria were listed as following: case-control studies, original data (independence among studies), and enough data to calculate an odds ratio (OR) with 95% confidence interval (CI). We excluded the following: studies in which the number of risk alleles could not be ascertained or contained overlapping data. These studies were assessed by two investigators independently. In case of any disagreement, it could be resolved by consultation with a third author.

### 2.3. Data Extraction

We extracted the following information from each article: first author, year of publication, demographics, ethnicity, the numbers of cases and controls, and allele frequency of the STAT4 rs7574865 polymorphism.

### 2.4. Statistical Analyses

We performed meta-analyses to estimate the association between STAT4 rs7574865 polymorphisms and SLE risk by odds ratios (ORs) and its 95% confidence interval (CI). The significance of the pooled ORs was determined by the *Z*-test, and *P* < 0.5 was considered statistically significant. In addition, Cochran^,^ s *Q* statistics was used to assess within-study and between-study variation and heterogeneities. This heterogeneity test assesses the null hypothesis that all studies evaluated the same effect. The effect of heterogeneity was quantified using *I*^2^. In our meta-analysis, *P* < 0.10 for the *Q*-test and *I*^2^ more than 50% was considered that significant heterogenicity among studies could not be ignored. Data were combined using both the fixed effects model (the inverse variance-weighted method) and the random effects model (DerSimonian and Laird method) [27, 28]. We carried out the random effects model when the effects are assumed heterogeneous in our study. All analyses were performed using the STATA 15.1 software.

### 2.5. Evaluation of Heterogeneity and Publication Bias

Subgroup analyses were performed by ethnicity, to examine the potential sources of heterogeneity observed in the meta-analyses. Because of the limitations of funnel plotting, publication bias was evaluated using the Egger linear regression test [29], which measures funnel plot asymmetry using a natural logarithm scale of ORs. We carried out sensitivity analysis to evaluate the effect of individual study on pooled OR. “Trim and fill” method was used to adjust summary estimates for observed bias [30], when asymmetry was indicated. In this method, small studies were removed until symmetry is achieved in the funnel plot by recalculating the center of the funnel, and then removed studies are replaced with their missing mirror-image counterparts. Finally, the revised summary estimate was calculated using all the original and hypothetical “filled” studies.

## 3. Result

### 3.1. Literature Search and Characteristics of Eligible Studies

**Figure 1:**
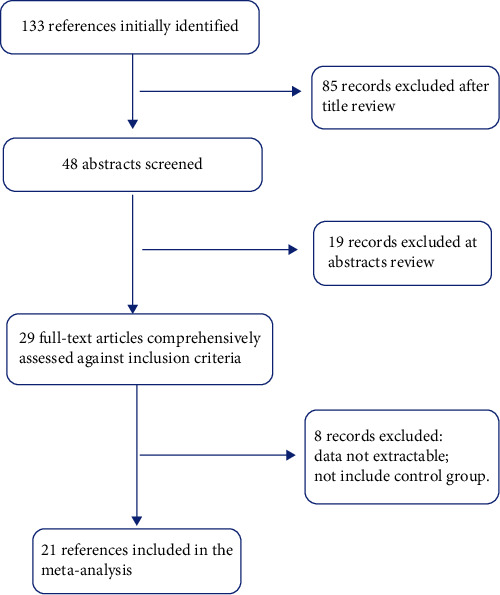
Flow chart of literature screening.

**Figure 2:**
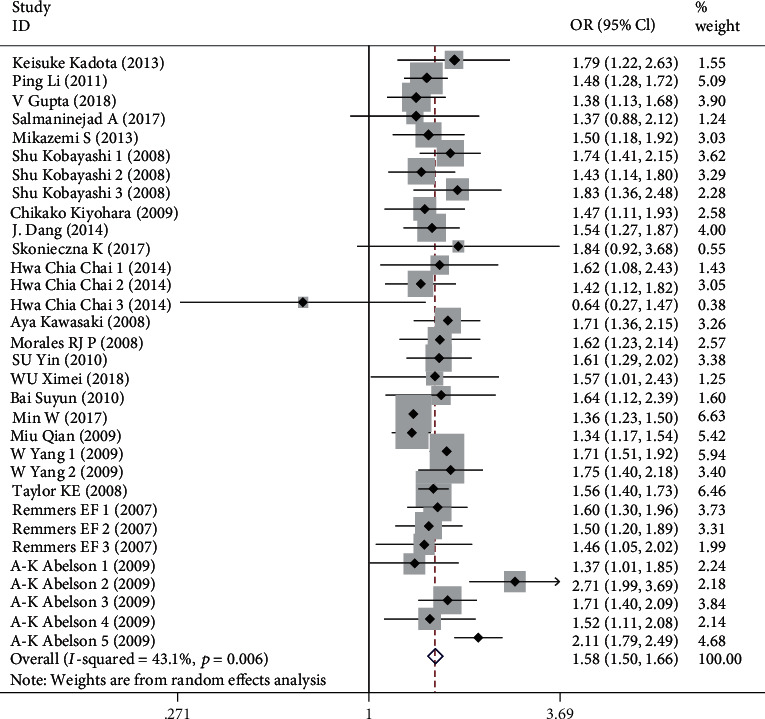
Forest plot for meta-analysis of STAT4 rs7574865 polymorphism and systemic lupus erythematosus (SLE) in all participants.

Figure 1 showed the literature searching process. 133 references were found by electronic and manual searches, of which 104 records were excluded by means of reading the title and abstract. Therefore, 29 full-text publications were judged potentially relevant, comprehensively assessed against inclusion criteria. Ultimately, 21 articles were included in our meta-analysis [6–26], excluding 8 studies with duplicate data and no case controls (Figure 1). Of these, one study contained data from five different groups [26], three studies contained data from three different groups, respectively [11, 15, 25], and one study contained data from two different groups [23]. These groups were analyzed independently, of which a total of 32 separate comparisons were considered, including 11384 SLE patients and 17609 controls. The major characteristics of individual eligible article were listed in Table 1 and Figure 2.

### 3.2. Frequency of the STAT4 rs7574865 T Allele in Different Ethnic Groups

As shown in Table 2, Asian had the greatest prevalence of STAT4 rs7574865 T allele and Europeans with lower prevalence. The mean frequency of T allele was 29.5%, and it varied from 21.8% to 32.2% in controls.

### 3.3. Meta-Analysis of the Association between the Polymorphism of STAT4 rs7574865 and SLE

**Figure 3:**
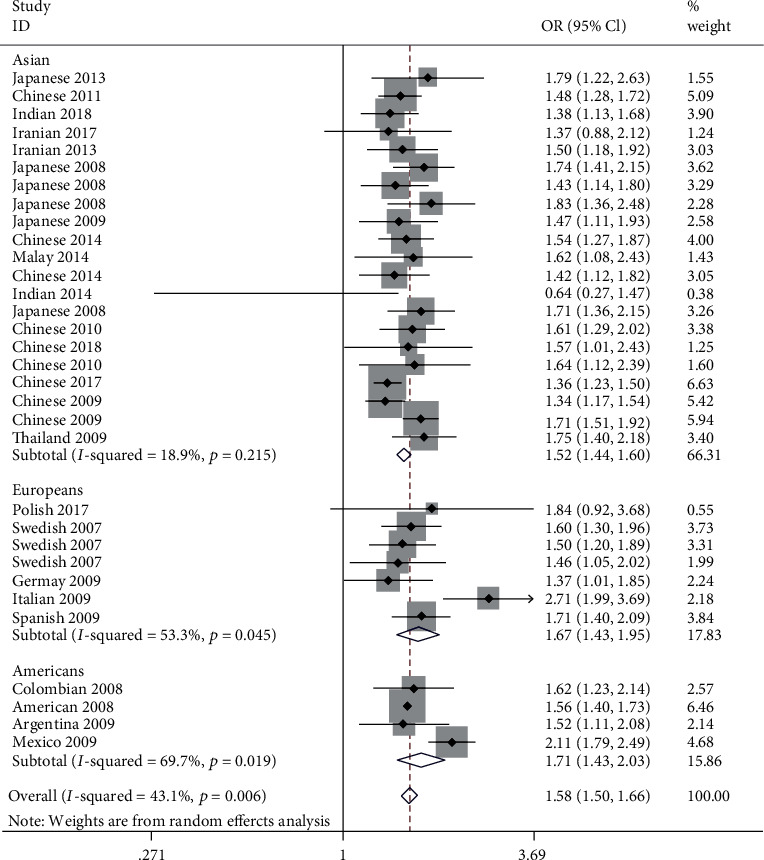
Forest plot for meta-analysis of STAT4 rs7574865 polymorphism and SLE in each ethnic group.

In our meta-analysis, the STAT4 rs7574865 T allele was shown strongly positive association with SLE (OR = 1.579, 95%CI = 1.497-1.665, *P* < 0.001) in all participants. The heterogeneity among studies was moderately obvious (*I*^2^ = 43.1%, *P* < 0.05). Thus, we performed stratified analysis by ethnicity, indicating that the association between risk factor T allele carriers and SLE was significant in Asians, Europeans, and Americans, with no obvious difference. The pooled ORs for the T allele of STAT4 rs7574865 were 1.706 (95%CI = 1.431-2.034, *P* < 0.001) for populations of American, 1.518 (95% CI = 1.440-1.600, *P* < 0.001) for Asian, 1.674 (95% CI = 1.433-1.955, *P* < 0.001) for Europeans. The results of subgroup analysis revealed that between-study heterogeneity was moderately obvious in European (*I*^2^ = 53.3%, *P* < 0.05) and American (*I*^2^ = 69.7%, *P* < 0.05), but no such heterogeneity was found in Asian (*I*^2^ = 18.9%, *P* > 0.05). (Table 3, Figure 3).

### 3.4. Publication Bias and Sensitivity Analyses

**Figure 4:**
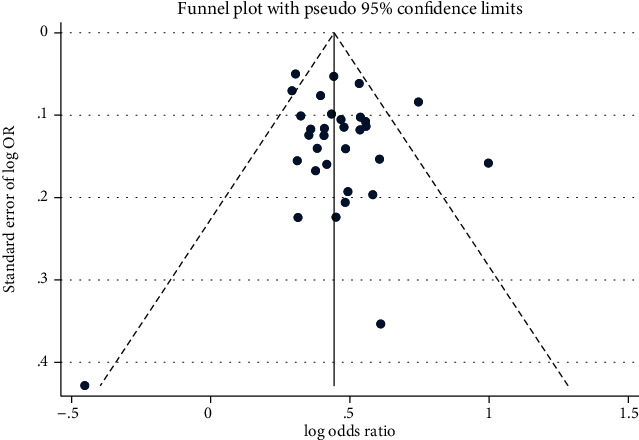
Publication bias for the analysis of association of STAT4 rs7574865 polymorphism and SLE susceptibility in overall populations.

We performed Egger's regression model and funnel plot to explore the potential publication bias of literatures, which results revealed no evidence of publication bias in individual groups (Egger regression *P* value > 0.05). (Table 3, Figure 4).

**Figure 5:**
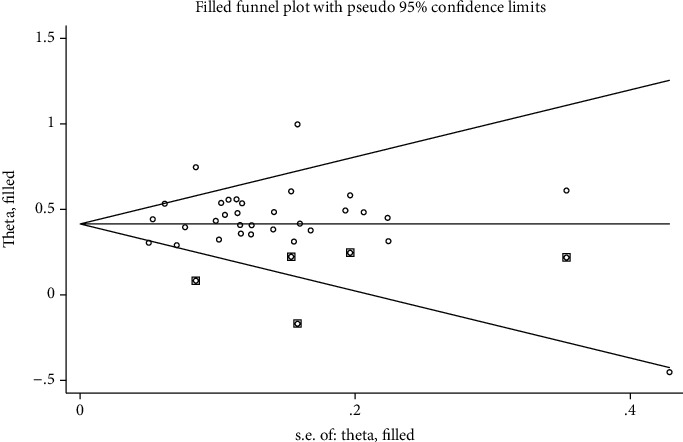
Funnel plots were obtained by trim and fill method to identify and correct the asymmetry of funnel plots caused by publication bias.

Sensitivity analysis was conducted to evaluate the influence of a single study on pooled OR by the “trim and fill” method. Corresponding pooled OR was not substantially changed when any individual study was deleted, which confirmed the stability of our overall result. The estimated number of missing studies is 5, and the adjusted pooled OR estimated is 1.514 (95%CI = 1.462-1.567), which showed still significantly increased risk for SLE (Figure 5).

## 4. Discussion

Systemic lupus erythematosus is a highly heterogeneous autoimmune disease, with influence of complex genetic. Studies in multiple ethnic populations have reported up to 100 risk loci of importance in SLE, although the mechanisms underlying these associations are still elusive [31–33].

Previous studies suggest that several single-nucleotide polymorphisms (SNPs) in LD in the third intron of STAT4, tagged by rs7574865, were initially demonstrated as SLE risk variants [25]. The minor T allele of rs7574865 is strongly linked with SLE [16]. Moreover, the STAT4 risk allele is also associated with specific clinical manifestations including earlier age at diagnosis, presence of anti-dsDNA, ischemic cerebrovascular disease, nephritis, and severe renal insufficiency [24, 34–36]. The above phenomena were consistent with the recent study which highlighted the role of STAT4 polymorphism in susceptibility to SLE [37]. However, due to the heterogeneity among different ethnic groups and a small sample size, existing evidences were not consistent.

In our comprehensive meta-analysis, we identified 21 relevant studies including 11384 SLE cases and 17609 healthy controls. The results do support a trend of association between STAT4 rs7574865 polymorphism and SLE risk in overall. When stratified by ethnicity for STAT4 rs7574865 polymorphism, positive correlations were observed in Asian, European, or American origin. Regarding STAT4 rs7574865 polymorphism in overall samples, we observed that the average frequency of T allele was 39.4% in patients with SLE compared with 29.5% in controls. The prevalence of the rs7574865 T allele was found to vary among ethnic populations.

Nevertheless, several limitations presented in this meta-analysis should be considered. First of all, the association between the STAT4rs7574865 polymorphism and disease severity and/or clinical features could not be performed limitation of amount of available data. Secondly, our ethnicity-associated results are applicable only to Asian, European, and American patients, as the data obtained from these ethnic groups. In addition, the heterogeneity has been presented obviously in European and American population, in subgroup analysis. Due to population demographics and lower SLE risk predisposition, or publication bias, fewer relevant studies were obtained in European and American, which may have distorted the heterogeneity, even have affected the final result, despite performing the “trim and fill” method and Egger regression test. Last but not least, STAT4 polymorphism may interact with other potential confounding risk factors.

In summary, our results stressed the importance of STAT4rs7574865 polymorphisms in increasing the risk of SLE in multiple ethnic groups and that the prevalence of the STAT4rs7574865 T allele may depend on ethnicity, which may improve our understanding of SLE pathogenesis. However, additional research with well-designed and large sample sizes is needed to be carried out about European, American, and other ethnicities in the future.

## Figures and Tables

**Table 1:** Characteristics of individual studies included in meta-analysis.

First author	Time	Ethnicity	Number		T allele (%)		Association	
			SLE	Control	SLE	Control	OR	95% CI
Keisuke Kadota	2013	Japanese	75	190	47.3	33.4	1.790	1.218-2.631
Ping Li	2011	Chinese	748	750	41.0	32.2	1.484	1.278-1.723
V Gupta	2018	Indian	394	583	32.9	26.2	1.382	1.134-1.685
Salmaninejad A	2017	Iranian	50	281	39.0	31.9	1.368	0.882-2.123
Mirkazemi S	2013	Iranian	280	281	41.3	31.9	1.502	1.176-1.918
Shu Kobayashi	2008	Japanese	238	752	44.0	31.0	1.744	1.411-2.154
		Japanese	188	940	39.0	31.0	1.432	1.139-1.800
		Japanese	165	212	44.0	30.0	1.833	1.357-2.476
Chikako Kiyohara	2009	Japanese	152	427	37.5	29.0	1.466	1.113-1.931
J. Dang	2014	Chinese	370	576	40.1	30.9	1.543	1.271-1.872
Skonieczna K	2017	Polish	35	50	32.9	21.0	1.841	0.921-3.681
Hwa Chia Chai	2014	Malay	93	110	44.1	32.7	1.621	1.082-2.427
		Chinese	245	294	46.3	37.8	1.423	1.115-1.815
		Indian	22	26	31.8	42.3	0.636	0.275-1.474
Aya Kawasaki	2008	Japanese	308	306	46.3	33.5	1.709	1.357-2.153
Morales RJ P	2008	Colombian	144	410	42.7	31.5	1.624	1.232-2.140
SU Yin	2010	Chinese	252	497	40.3	29.5	1.614	1.289-2.019
WU Xi-mei	2018	Chinese	163	102	50.3	35.3	1.568	1.012-2.431
Bai Suyun	2010	Chinese	102	125	47.1	35.2	1.636	1.121-2.388
Min W	2017	Chinese	1387	2259	40.3	33.2	1.356	1.229-1.495
Miu Qian	2009	Chinese	767	956	42.8	35.8	1.338	1.166-1.536
W Yang,	2009	Chinese	910	1440	46.1	33.4	1.705	1.512-1.923
		Thailand	278	383	48.7	35.2	1.747	1.398-2.183
Taylor KE	2008	American	1398	2560	31.1	22.5	1.556	1.403-1.726
Remmers EF	2007	Swedish	575	416	31.0	22.0	1.597	1.299-1.962
		Swedish	349	416	31.0	23.0	1.504	1.198-1.889
		Swedish	115	416	29.0	22.0	1.458	1.050-2.024
A-K Abelson	2009	Germany	247	220	26.5	21.0	1.365	1.007-1.851
		Italy	221	207	39.4	19.3	2.711	1.989-3.695
		Spain	390	620	32.7	22.1	1.712	1.401-2.094
		Argentina	171	171	41.5	31.9	1.518	1.110-2.076
		Mexico	552	633	54.7	36.4	2.109	1.789-2.487
Total			11384	17609	39.4	29.5	1.579	1.497-1.665

CI: confidence interval; OR: odds ratio.

**Table 2:** Prevalence of T allele for STAT4 rs7574865 polymorphism in SLE patients and controls of each ethnicity.

Population	No. of studies	Numbers		T allele (%)	
		SLE	Control	SLE	Control
Asian	21	7187	11490	41.7	32.2
European	7	1932	2345	31.7	21.8
American	4	2265	3774	38.4	26.2
Overall	32	11384	17609	39.4	29.5

**Table 3:** Comparison of the association between the STAT4 rs7574865 polymorphism and SLE in each ethnicity.

Population	No. of studies	Test of association			Test of heterogeneity		Publication bias (Egger's test)
		OR	(95% CI)	*P* value	*I* ^2^ (%)	*P* value	*P* value
Asian	21	1.518	1.440-1.600	<0.001	18.9	0.215	0.517
European	7	1.674	1.433-1.955	<0.001	53.3	0.045	0.731
American	4	1.706	1.431-2.034	<0.001	69.7	0.019	0.827
Overall	32	1.579	1.497-1.665	<0.001	43.1	0.006	0.457

## Data Availability

The extracted data used to support the findings of this study are mainly included within the article.
